# Correspondence

**Published:** 1871-03

**Authors:** John Hauenstein


### Correspondence.

*Editor of the Buffalo Medical Journal:*

Dear Sir,—Permit me, through the colums of your *Journal* to call the attention of the medical profession to an enterprise which, of late years, has become so flourishing that, with a little more experience of the persons engaged in it, threatens to become a *big swindle* upon the tax-payers of Erie county. It can, already, with propriety, be called a flagrant abuse of a high official trust. I allude to the multiplicity of unnecessary coroner's inquests, of which the following is an example:

I was called to see a patient at No. 162 Pratt street, late on the evening of Feb. 27th. I found him setting up in a chair, and upon inquiry learned that he was about fifty-seven years of age; and for the two years last passed, had not been occupied in any capacity except that of a *hard drinker*. An examination revealed inflammation of the right lung, characterized by dullness of percussion, crepitant rale, bronchopony, &c., together with the rusty expectoration, the pathognomonic sign of pneumonia. Respiration about 40, pulse 136 to 140. Insomuria, with occasional attacks of delirium, such as we find in delirium tremens, but with long intervals of being perfectly rational.

The gravity of the symptoms varied but little, until March 2d, the fourth day of my attendance, when it became evident that the patient must die. I then suggested to him the propriety of making his last will and testament as to his property, if any he had which he consented to do, and *did do* that same day, he being quite rational. The next day, the fifth of my attendance on him, I found my patient dead. In a fit of delirium he imagined that he must go back to the pig-sty to prevent the escape of the pigs, which he thought were very boisterous; and notwithstanding the protestation of his daughter-in-law, the only person in attendance in the house, he went out, and on coming into the house again, he was so exhausted that he fell, and died in a few minutes, upon the floor of the kitchen. When I returned to my office I filled out a certificate of death, "died of inflammation of the lungs, hastened by intemperance," and placed it in a drawer to which the undertaker has access, and where it lies yet.

Under the circumstances, I considered it a very natural death and one not at all unexpected; but when, on Monday following, March 6th, the son of the deceased came to my office to pay me for the services rendered, and told me that the coroner had summoned a jury, and had held an inquest on the body of his father; I was not a little surprised, and for the moment apprehending that possibly some one had informed the coroner of malpractice, neglect of duty, or something else, on my part, I hastened to find out the verdict in order to make good my escape from the clutches of the police. I was not long in finding the little article in the newspaper so familiar to the reader of local news, and to my great relief I saw that I was not accused of killing the man; but that he had died of that stereotyped disease, which is the fate of all those on whom the coroner may chance to smile, and which he gravely calls "disease of the heart." Further comments I leave to you.

Respectfully Yours,

JOHN HAUENSTEIN.

March 14th, 1871.

